# Association of Attenuated Plaques Detected by Intravascular Ultrasound With Plaque Calcification Assessed by Computed Tomography Angiography

**DOI:** 10.31083/RCM45291

**Published:** 2026-01-21

**Authors:** Yang Zhao, Jiaying Li, Wenxuan Dou, Jingyao Yuan, Xin Huang

**Affiliations:** ^1^Department of Cardiology, The First Affiliated Hospital of Xi’an Jiaotong University, 710061 Xi’an, Shaanxi, China

**Keywords:** attenuated plaque, coronary artery calcium, intravascular ultrasound, computed tomography angiography, calcium density, plaque stability

## Abstract

**Background:**

Coronary artery calcium (CAC) reflects the overall atherosclerotic burden. The CAC density is inversely associated with plaque vulnerability. Intravascular ultrasound (IVUS)-defined attenuated plaques represent unstable lesions, which are linked to adverse clinical outcomes. Meanwhile, the determination as to whether coronary computed tomography angiography (CCTA)-derived CAC metrics can serve as noninvasive markers of attenuated plaques remains uncertain.

**Methods:**

This retrospective study included coronary artery disease (CAD) patients who underwent both CCTA and IVUS between January 2023 and December 2024 at our medical center. CCTA was used to quantify plaque volume, density, and composition (lipid, fiber, and calcium), while IVUS was employed to characterize the plaques as attenuated and non-attenuated.

**Results:**

Among 94 patients with 150 coronary plaques, calcium volume showed a very strong correlation with total plaque volume (*r*_s_ = 0.953, *p* < 0.0001). Meanwhile, attenuated plaques exhibited significantly lower calcium density (321.00 vs. 499.00 Hounsfield units (HU); *p* = 0.0004), calcium volume (55.20 vs. 168.10 mm^3^; *p* = 0.003), and calcium percentage (33.30% vs. 55.40%; *p* = 0.015) compared with the non-attenuated plaques. Multivariate logistic regression analysis identified lower CAC density as the only independent predictor of IVUS-confirmed attenuated plaques (odds ratio = 0.994, 95% confidence interval (CI): 0.990–0.997; *p* = 0.0002). The area under the receiver operating characteristic (AUROC) curve for CAC density in diagnosing attenuated plaques was 0.735 (95% CI: 0.603–0.868; *p* = 0.0004). At a cutoff of 461.50 HU, the sensitivity and specificity were 81.8% and 66.1%, respectively.

**Conclusion:**

CCTA-derived CAC volume reflects the atherosclerosis (AS) burden, while lower CAC density independently predicts IVUS-confirmed attenuated plaques. A higher CAC density was associated with plaque stability, suggesting that the CCTA-derived CAC density may serve as a noninvasive marker of plaque stability, aiding in the assessment of plaque vulnerability and risk stratification.

## 1. Introduction

Coronary artery calcium (CAC) serves as a surrogate marker for overall atherosclerotic burden and is strongly linked to an elevated risk of plaque rupture and all-cause mortality [1, 2]. High CAC scores are associated with a 10-fold increase in acute coronary events and a 4-fold increase in death [1, 2]. While CAC volume shows positive and independent correlations with coronary artery disease (CAD) and cardiovascular risk factors, CAC density displays an inverse association with plaque vulnerability at any level of CAC volume [3, 4].

Attenuated plaque on Intravascular ultrasound (IVUS) is characterized by hypoechoic regions with deep ultrasonic attenuation despite lacking bright calcium in the coronary atherosclerotic plaques [5]. Prior studies have shown that densely calcified plaques progress more slowly than spotty plaques, whereas IVUS-defined attenuated plaques correlate with procedural complications during percutaneous coronary intervention (PCI) and future cardiovascular events [5, 6]. Contemporary histopathologic and imaging evidence confirms that high CAC density is characteristic of stable fibrocalcific plaques and inversely correlates with necrotic core dimensions [1, 7]. These findings suggest that integrating coronary computed tomography angiography (CCTA)-derived CAC metrics with IVUS-defined plaque features could improve non-invasive risk assessment.

Therefore, this study aimed to investigate the relationship between CCTA-derived CAC volume and density and IVUS-defined attenuated plaques in patients with CAD, to evaluate their potential as non-invasive markers for plaque vulnerability and risk stratification.

## 2. Methods

### 2.1 Study Design and Patients

A retrospective review was performed on consecutive patients who received clinically indicated CCTA, followed by elective IVUS evaluation for newly developed coronary artery plaques, at the First Affiliated Hospital of Xi’an Jiaotong University during the period from January 2023 to December 2024. Exclusion criteria included a CCTA–IVUS interval longer than four weeks, suboptimal CCTA imaging, or previous revascularization of the lesions under investigation. Ethical approval was obtained from the Ethics Committee of the First Affiliated Hospital of Xi’an Jiaotong University (XJTU1AF2019LSL-014), and the study adhered to the principles of the Declaration of Helsinki.

Data were retrospectively collected, including baseline information, clinical manifestations, cardiovascular risk factors, angiography results, and quantitative CCTA/IVUS measurements.

### 2.2 CCTA Image Acquisition

CCTA was performed on a Revolution computed tomography (CT) scanner (GE Healthcare, Shanghai, China) using retrospective electrocardiography-gated tube current modulation. The imaging parameters included: slice collimation 256 × 0.625 mm; gantry rotation time 270 ms; tube voltage 80–120 kVp; and automated choice of mAs value based on patient weight. A double-head power injector (Ulrich Medical AG, Ulm-Jungingen, Germany) was used to inject contrast media through a 20G trocar in an antecubital vein. A weight-dependent bolus of 70–90 mL iodine contrast agent (iohexol; GE Healthcare, Shanghai, China) was injected at a rate of 4 to 5.5 mL/s, followed by a 30-mL saline flush. Image reconstruction was performed at 45% (mid-diastole) and 75% (end-systole) of the cardiac cycle using iterative reconstruction.

### 2.3 CCTA Image Analysis

The CCTA database was independently reviewed on a Vitrea workstation (Vital Images, Minnetonka, MN, USA) by two observers who were blinded to clinical information and each other’s assessments. Final measurements were derived from the average of both readings.

Plaques were defined as structures larger than 1 mm^2^ located within or adjacent to the vessel lumen and clearly identified from both the lumen and surrounding pericardial tissue. Plaque borders were manually adjusted when necessary. The mean plaque burden was calculated as the ratio of total plaque volume to total vessel volume, expressed as a percentage. The remodeling index (RI) was calculated as the ratio of the cross-sectional area at the lesion site to that of the proximal reference segment, with RI ≥1.1 considered to be positive remodeling. Plaque composition was quantified using established Hounsfield unit thresholds (HU): –100 to 49 HU for lipid; 50 to 149 HU for fiber, and 150 to 1300 HU for calcium. The absolute volumes (mm^3^) and relative percentages of each component were automatically computed from all voxels within the selected coronary segment.

### 2.4 IVUS Image Acquisition and Analysis

The IVUS catheter (40 MHz Opticross, Boston Scientific, Marlborough, MA, USA) was advanced along the guide wire, and image acquisition was carried out using the iLab ultrasound imaging system (Boston Scientific, Marlborough, MA, USA). Images were recorded during automatic pullback at a speed of 0.5 mm/s. Off-line analyses were conducted with dedicated software (QIvus 3.0, Medis medical imaging systems, Leiden, the Netherlands) with consensus by two independent observers who were blinded to other image results from the same patient. Attenuated plaques were defined as hypoechoic lesions showing marked ultrasound attenuation in the absence of calcification or very dense fibrotic tissue [8].

### 2.5 Statistical Analysis

Continuous variables are presented as median (IQR) as they were non-normally distributed. Categorical variables were summarized as counts and percentages. For group comparisons, Student’s *t*-test or the Mann-Whitney U test was used for continuous data, while categorical variables were analyzed using the chi-square test or Fisher’s exact test. Correlations between calcium density, volume, and the total plaque volume were analyzed using Spearman correlations (*r*_s_). Correlation coefficients of <0.2 were regarded as very weak, 0.2 to <0.40 as weak, 0.40 to <0.60 as moderate, 0.6 to <0.80 as strong, and 0.8 to 1 as very strong. Binary logistic regression was used to evaluate the association between calcium density and attenuated plaque. Receiver operating characteristic (ROC) curve analysis evaluated the predictive performance, with optimal cut-off point determined by the maximum Youden index. Statistical significance was defined as a two-sided *p* < 0.05. Statistical analyses and data visualization were performed using IBM SPSS Statistics (version 26.0; IBM Corp., Armonk, NY, USA).

## 3. Results

### 3.1 Characteristics of the Study Population

**Fig. 1.**
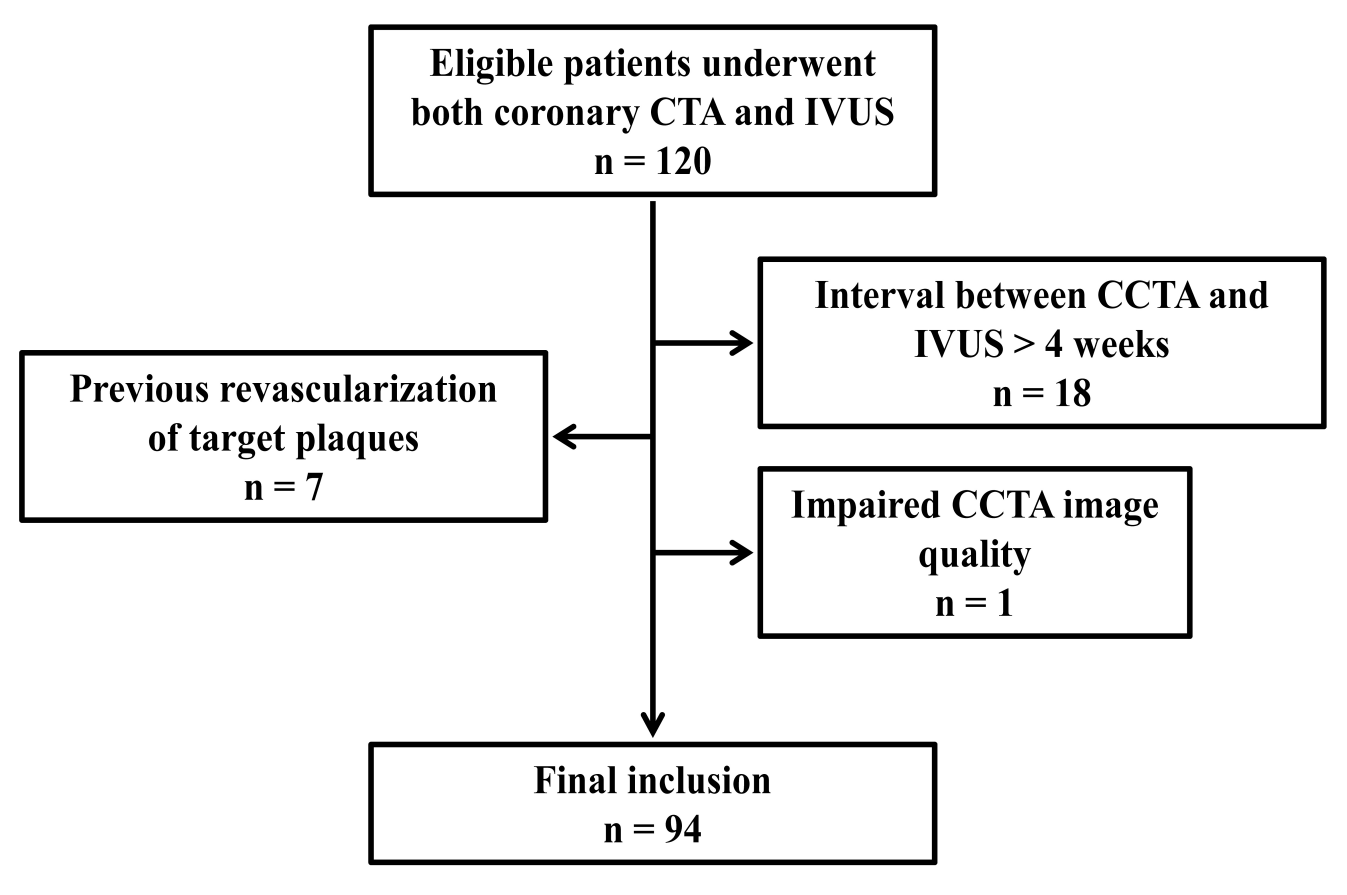
**Flowchart of the study population**. CTA, computed tomography angiography; CCTA, coronary computed tomography angiography; IVUS, intravascular ultrasound.

From January 2023 to December 2024, 120 patients who underwent both CCTA and IVUS were initially reviewed. Eighteen patients were excluded due to an interval between CCTA and IVUS exceeding 4 weeks, one was excluded because of impaired CTA image quality, and seven were excluded due to prior revascularization of the target lesion (Fig. 1). The final study consisted of 94 patients with 150 lesions. The median age of the study population was 63.0 years (IQR: 54.0–70.30), and 78.7% (n = 74) were male (Table 1). Patients were stratified into two groups based on IVUS measurements: (1) the attenuated plaque group (patients with ≥1 plaque meeting IVUS attenuation criteria), and (2) the non-attenuated plaque group. Baseline characteristics and cardiovascular risk factors showed no significant differences between the two groups (Table 1).

**Table 1.** **Baseline Characteristics of the study population**.

		Whole cohort (n = 94)	Attenuated (n = 15)	Non-attenuated (n = 79)	*p* value
Variables					
	Age (year), median (IQR)	63.0 (54.0–70.3)	60.0 (54.0–67.0)	63.0 (54.0–71.0)	0.559
	Men (%)	74 (78.7)	13 (86.7)	61 (77.2)	0.513
	BMI (kg/m^2^), median (IQR)	24.9 (23.7–26.7)	24.9 (23.4–27.2)	24.9 (23.7–26.7)	0.732
	Current smoking (%)	23 (24.5)	3 (20.0)	20 (25.3)	1.000
	Diabetes (%)	35 (37.2)	7 (46.7)	28 (35.4)	0.410
	Hypertension (%)	68 (72.3)	10 (66.7)	58 (73.4)	0.753
	Dyslipidemia (%)	94 (100)	15 (100)	79 (100)	NC^1^
	Prior MI (%)	20 (21.3)	3 (20.0)	17 (21.5)	1.000
	Prior PCI (%)	20 (21.3)	2 (13.3)	18 (22.8)	0.513
	Chronic renal failure (%)	8 (8.5)	1 (6.7)	7 (8.9)	1.000
	Family history of CAD (%)	9 (9.7)	1 (10.3)	8 (6.7)	1.000
Clinical presentation					
	Stable AP (%)	16 (17.0)	3 (20.0)	13 (16.5)	0.715
	ACS (%)	78 (83.0)	12 (80.0)	66 (83.5)	0.715
	TCHO (mmol/L), median (IQR)	3.4 (2.9–4.1)	3.5 (3.0–4.0)	3.3 (2.9–4.3)	0.800
	HDL-C (mmol/L), median (IQR)	0.9 (0.7–1.0)	0.8 (0.8–1.1)	0.9 (0.7–1.0)	0.938
	LDL-C (mmol/L), median (IQR)	2.0 (1.5–2.6)	2.0 (1.6–2.4)	2.0 (1.5–2.6)	0.955
	TG (mmol/L), median (IQR)	1.4 (1.0–1.9)	1.2 (0.9–1.5)	1.4 (1.0–2.0)	0.302
	Lipoprotein a (mg/L), median (IQR)	213.5 (130.0–340.0)	239.0 (86.0–337.0)	209.0 (138.0–349.0)	0.624
	Hemoglobin A1C (%), median (IQR)	6.1 (5.6–6.8)	6.0 (5.3–7.0)	6.1 (5.6–6.7)	0.804
	FBG (mmol/L), median (IQR)	5.7 (5.1–7.1)	6.1 (4.9–8.3)	5.6 (5.1–7.1)	0.421
	GFR (mL/min), median (IQR)	106.1 (86.5–119.2)	97.0 (87.5–117.8)	106.8 (85.4–121.1)	0.389
	hs-cTnT (ng/mL), median (IQR)	0.01 (0.006–0.0435)	0.008 (0.005–0.068)	0.011 (0.006–0.039)	0.535
	pro-BNP (pg/mL), median (IQR)	131.9 (53.5–705.5)	230.2 (65.7–540.0)	125.2 (52.0–912.8)	0.769
	hs-CRP (mg/L), median (IQR)	2.8 (1.1–2.9)	2.6 (0.9–2.9)	2.9 (1.2–2.9)	0.389
	LVEF (%), median (IQR)	67.0 (61.0–71.0)	67.0 (61.0–70.0)	66.0 (61.0–71.0)	0.975
	Multivessel disease (%)	66 (70.2)	11 (73.3)	55 (69.6)	1.000

^1^The *p*-value for dyslipidemia prevalence between groups was not computed (NC) due to identical rates (100%) in all subgroups. Continuous variables in this table are presented as median (IQR) as they were non-normally distributed. Categorical variables are summarized as counts and percentages. BMI, body mass index; MI, myocardial infarction; PCI, percutaneous coronary intervention; CAD, coronary heart disease; AP, angina pectoris; ACS, acute coronary syndrome; TCHO, total cholesterol; HDL-C, high-density lipoprotein cholesterol; LDL-C, low-density lipoprotein cholesterol; TG, triglyceride; FBG, fasting blood glucose; GFR, glomerular filtration rate; pro-BNP, pro-brain natriuretic peptide; hs-CRP, high-sensitivity C-reactive protein; LVEF, left ventricular ejection fraction; hs-cTnT, high-sensitivity cardiac troponin T.

### 3.2 Characteristics of IVUS-Defined Attenuated and Non-attenuated Plaques

Among 150 analyzed plaques, 14.7% (n = 22) were identified as attenuated plaques by IVUS and 85.3% (n = 128) as non-attenuated plaques. Comparison of angiography and CCTA characteristics between the attenuated and non-attenuated plaques is illustrated in Table 2.

**Table 2.** **Comparison between the attenuated and non-attenuated plaques**.

Variables			Attenuated (n = 22)	Non-attenuated (n = 128)	*p* value
Angiography outcomes					
	Lesion location (%)				
		LAD	14 (63.6)	69 (53.9)	0.396
		LCX	6 (27.3)	35 (27.3)	0.995
		RCA	2 (9.1)	24 (18.8)	0.369
	Initial TIMI flow grade 0–1		1 (4.5)	21 (16.4)	0.201
	Moderate to heavy calcification on angiography		4 (18.20)	56 (43.75)	0.024
	Type B2/C lesion		14 (63.6)	76 (59.4)	0.706
Quantitative computed tomography angiography analysis					
	Minimal lumen diameter (mm), median (IQR)		0.90 (0.10–3.37)	0.90 (0.30–2.52)	0.067
	Reference vessel diameter (mm), median (IQR)		3.50 (3.18–3.80)	3.42 (2.98–3.91)	0.707
	Diameter stenosis (%), median (IQR)		85.50 (65.30–95.00)	82.25 (59.90–94.00)	0.059
	Lesion length (mm), median (IQR)		15.25 (11.20–27.23)	19.90 (13.90–33.75)	0.127
	Mean plaque burden (%), median (IQR)		69.55 (62.45–80.95)	77.65 (67.63–84.93)	0.066
	Total plaque volume (mm^3^), median (IQR)		183.00 (108.25–275.50)	299.00 (148.25–548.83)	0.018
	Lipid volume (mm^3^), median (IQR)		41.90 (24.60–77.80)	58.75 (36.20–96.40)	0.085
	Fiber volume (mm^3^), median (IQR)		61.35 (37.62–110.93)	87.15 (47.68–130.15)	0.120
	Calcium volume (mm^3^), median (IQR)		55.20 (14.48–139.05)	168.10 (56.20–319.05)	0.003
	Lipid percentage (%), median (IQR)		28.15 (16.98–34.20)	19.60 (15.40–25.33)	0.060
	Fiber percentage (%), median (IQR)		34.65 (25.15–52.73)	24.85 (19.63–34.48)	0.006
	Calcium percentage (%), median (IQR)		33.30 (14.00–56.60)	55.40 (39.98–63.98)	0.015
	Total density (HU), median (IQR)		115.50 (84.75–283.25)	293.50 (203.00–375.00)	0.002
	Lipid density (HU), median (IQR)		4.00 (–1.00–12.25)	–1.00 (–6.00–5.00)	0.008
	Fiber density (HU), median (IQR)		97.50 (94.00–100.25)	98.00 (96.00–100.00)	0.447
	Calcium density (HU), median (IQR)		321.00 (211.00–455.75)	499.00 (399.25–554.50)	0.0004

Continuous variables in this table are presented as median (IQR) as they were non-normally distributed. Categorical variables are summarized as counts and percentages. LAD, left anterior descending; LCX, left circumflex; RCA, right coronary artery; TIMI, thrombolysis in myocardial infarction; HU, Hounsfield units.

**Fig. 2.**
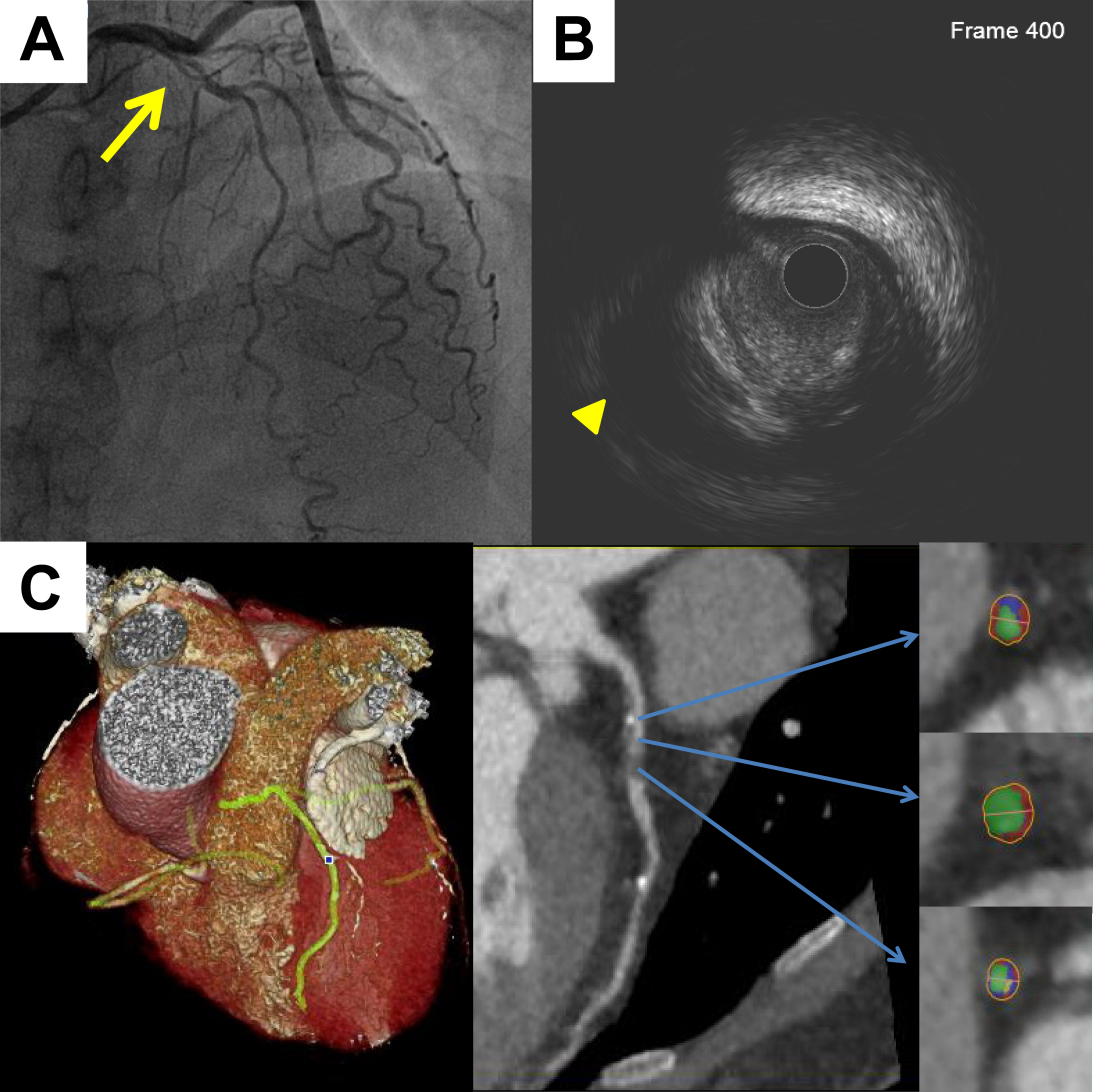
**A representative case of quantitative CCTA analysis of an attenuated plaque**. (A) Invasive angiography revealed intermediate coronary stenosis at the proximal left anterior descending artery (yellow arrow). (B) IVUS confirmed the presence of attenuated plaque (yellow triangle). (C) CCTA imaging demonstrated the attenuated plaque. In the color-coded cross-sectional images, lipid areas were displayed in red, fibrous tissue in blue, and calcified regions in yellow. CCTA, coronary computed tomography angiography; IVUS, intravascular ultrasound.

The attenuated plaques exhibited significantly less moderate to heavy calcification compared to non-attenuated plaques (18.20 % vs. 43.75%, *p* = 0.024). These attenuated plaques also demonstrated smaller total plaque volume (183.00 vs. 299.00 mm^3^, *p* = 0.018), lower calcium volume (55.20 vs. 168.10 mm^3^, *p* = 0.003), and reduced calcium percentage (33.30 % vs. 55.40%, *p* = 0.015), while containing a higher percentage of fibrous tissue (34.65 % vs. 24.85%, *p* = 0.006). Density analysis revealed that attenuated plaques had significantly lower total density (115.50 vs. 293.50 HU, *p* = 0.002), calcium density (321.00 vs. 499.00 HU, *p* = 0.0004), but a higher lipid density (4.00 vs. –1.00 HU, *p* = 0.008). No statistically significant differences were observed in minimal lumen diameter, reference vessel diameter, diameter stenosis and lesion length. Fig. 2 shows a representative case of quantitative CCTA analysis for an attenuated plaque.

### 3.3 Correlations of CCTA-Derived Quantitative Plaque Parameters With CAC

**Fig. 3.**
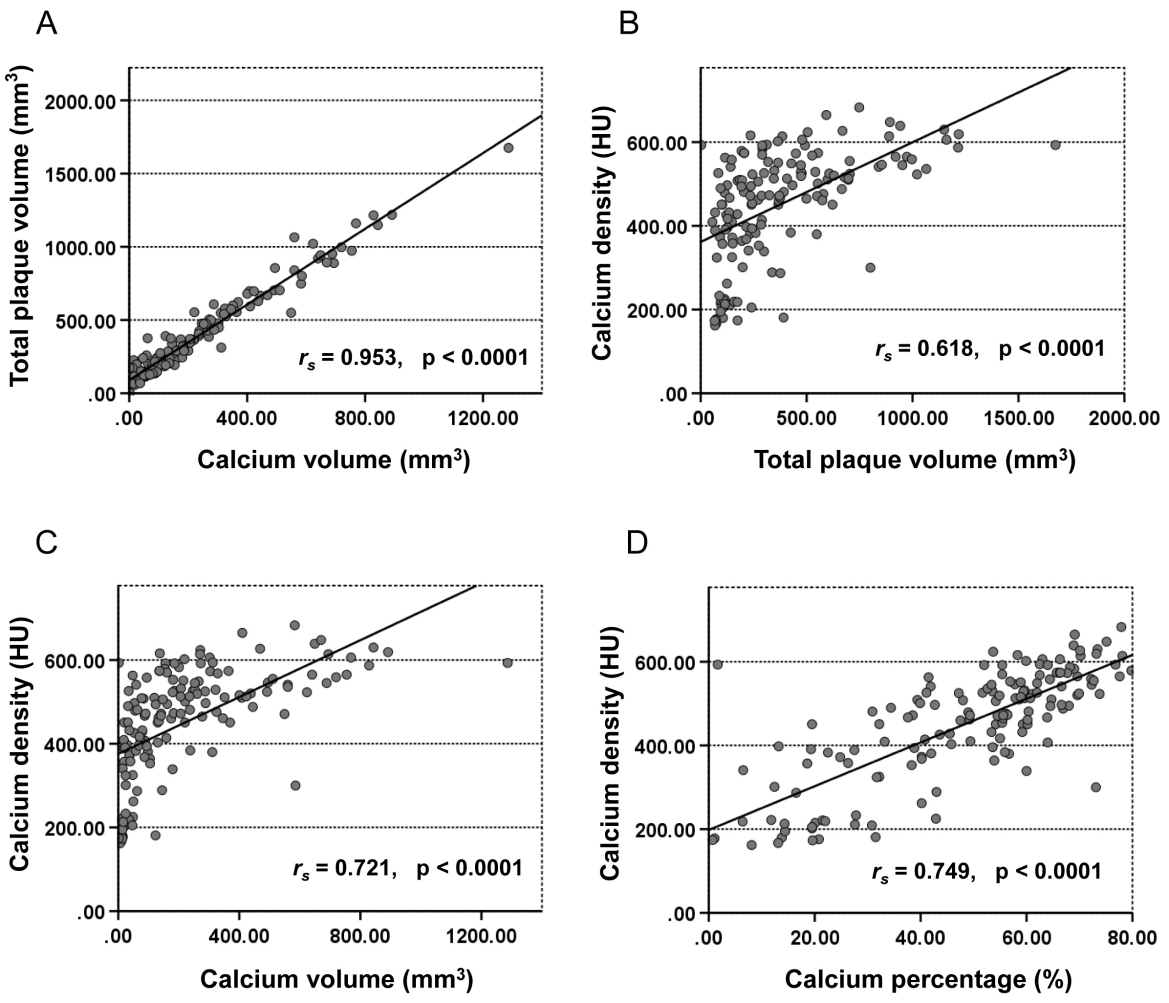
**Correlations between the CAC and the quantitative parameters as measured on computed tomography angiography**. (A) The calcium volume of plaques showed a strong correlation with total plaque volume (*r*_s_ = 0.953, *p* < 0.0001). (B) CAC density correlated strongly with the total plaque volume (*r*_s_ = 0.618, *p* < 0.0001); (C) with calcium volume (*r*_s_ = 0.721, *p* < 0.0001); (D) with the calcium percentage (*r*_s_ = 0.749, *p* < 0.0001). CAC, coronary artery calcium; HU, Hounsfield units.

Fig. 3 illustrates the correlations between CCTA-derived quantitative plaque parameters and CAC. Calcium volume exhibited a very strong positive correlation with total plaque volume (*r*_s_ = 0.953, *p* < 0.0001; Fig. 3A). Calcium density was strongly associated with total plaque volume *r*_s_ = 0.618, *p* < 0.0001; Fig. 3B), calcium volume (*r*_s_ = 0.721, *p* < 0.0001; Fig. 3C), and calcium percentage (*r*_s_ = 0.749, *p* < 0.0001; Fig. 3D). Lipid volume showed a moderate correlation with calcium density (*r*_s_ = 0.428, *p* < 0.0001). The volume of fiber demonstrated only a weak correlation with calcium density (*r*_s_ = 0.273, *p* = 0.001).

### 3.4 Diagnostic Performance of CAC Density for Predicting Attenuated Plaques

**Fig. 4.**
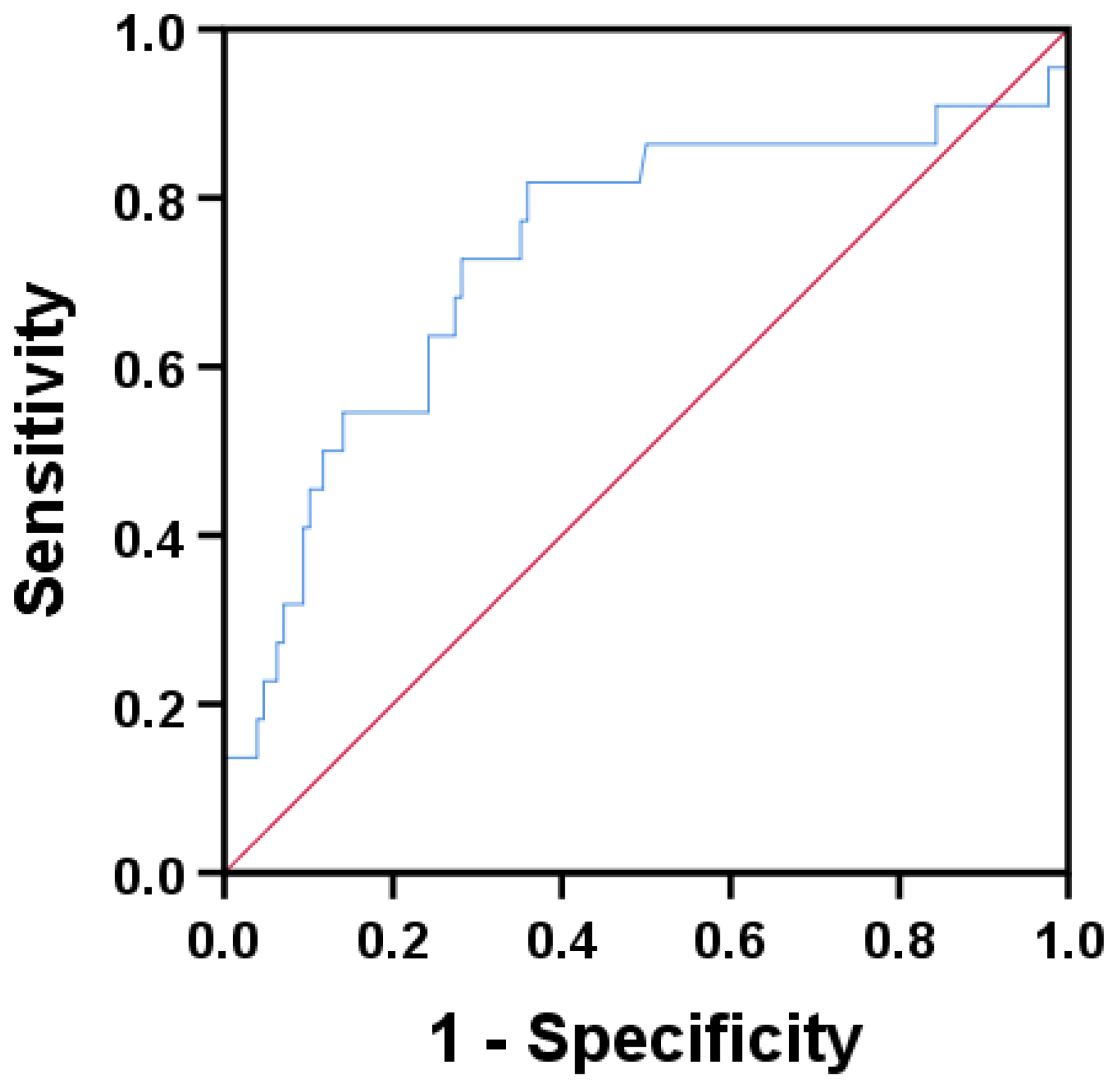
**ROC curve analysis of CAC density for the prediction of IVUS-derived attenuated plaques**. The AUC was 0.735 (95% CI: 0.603–0.868, *p* = 0.0004).

After adjusting for total plaque volume, lipid percentage, calcium percentage, and fiber percentage, calcium density remained an independent predictor for attenuated plaque (odds ratio [OR] = 0.994, 95% CI: 0.990–0.997, *p* = 0.0002). The AUC of CAC density to diagnose attenuated plaques was 0.735 (95% CI: 0.603–0.868, *p* = 0.0004, Fig. 4). Using a cutoff value of 461.50 HU, CAC density showed a diagnostic sensitivity of 81.8% and specificity of 66.1% for identifying attenuated plaques.

## 4. Discussion

The main findings of this study can be summarized as follows: (1) CAC volume demonstrated a strong correlation with total plaque volume, which reflected total atherosclerosis (AS) burden. (2) Lower CAC density detected by CCTA was an independent predictor for IVUS-confirmed attenuated plaques. (3) A CAC density above 461.5 HU was associated with plaque stability.

Prior studies have established that CAC occurs at all stages of AS and is highly specific for the disease [9]. The Agatston CAC score, based on non-contrast computed tomography scans of CAC volume and peak density, outperforms traditional risk scores in predicting events [10]. The MESA (Multi-Ethnic Study of Atherosclerosis) study found that a higher density of CAC conferred lower cardiovascular risk after adjusting for volume [3]. Similarly, van Rosendael AR *et al*. [11] demonstrated that very dense calcium (the so-called “1K Plaque”, i.e., >1000 HU) was associated with a lower risk of subsequent acute coronary events. Our results are consistent with these findings, showing that calcium volume and density correlate strongly with total plaque volume, reinforcing the role of CAC quantity and quality as robust CAD risk markers.

Attenuated plaque, an IVUS-derived feature of vulnerable plaques, is characterized by large plaque burden, microcalcification, thin-capped fibroatheroma with lipid-rich core, and macrophage infiltration surrounding a large necrotic core [5]. These features collectively contribute to plaque instability and a higher incidence of cardiovascular events [12].

In this analysis, we demonstrated that attenuated plaques exhibit significantly higher non-calcium component percentages but lower calcium percentages compared to non-attenuated plaques. After adjustment for total plaque volume, calcium percentage, fiber percentage and lipid percentage, lower CAC density remained an independent predictor of attenuated plaques. Furthermore, our analysis identified that the CAC density above 461.5 HU is associated with more stable lesions.

By directly comparing CCTA-derived CAC metrics with IVUS-defined attenuated plaques, our study bridges non-invasive and invasive imaging modalities. These results suggest that CAC density measured by CCTA may serve as a reliable non-invasive tool for the identification of high-risk plaques and for risk stratification in CAD. In addition, emerging artificial intelligence (AI) approaches may further advance this field by enabling automated and reproducible assessments, enhancing detection of high-risk features, and integrating multi-modality imaging data to improve diagnostic accuracy and strengthen the role of CCTA in non-invasive, patient-tailored risk stratification, ultimately supporting earlier intervention and more precise management of CAD [13, 14].

## 5. Limitations

This study has several limitations. First, it was a single-center retrospective study, and included a relatively small cohort, with only 22 attenuated plaques, which may limit statistical power and generalizability. Second, analyses were performed at the lesion level without accounting for intra-patient clustering, which may introduce bias. Third, reproducibility of attenuated plaque assessment was not evaluated. Finally, calcification morphology such as spotty or microcalcification, was not analyzed. Future multicenter studies with larger cohorts, reproducibility testing, and detailed assessment of calcification morphology are needed to validate and extend our findings.

## 6. Conclusion

CCTA-derived CAC volume is reflective of overall AS burden, while lower CAC density detected by CCTA was an independent predictor of IVUS-defined attenuated plaques. A CAC density above 461.5 HU was associated with plaque stability. These findings suggest that CCTA-derived CAC density could serve as a non-invasive marker of plaque vulnerability and risk stratification. Future multicenter studies with larger cohorts and AI-based approaches are necessary to validate and expand these results.

## Availability of Data and Materials

The data used in the current study are available from the corresponding author upon reasonable request.
